# Broccoli (*Brassica oleracea var. italica*) leaves exhibit significant antidiabetic potential in alloxan-induced diabetic rats: the putative role of ABC vacuolar transporter for accumulation of Quercetin and Kaempferol

**DOI:** 10.3389/fphar.2024.1421131

**Published:** 2024-12-11

**Authors:** Sara Latif, Muhammad Sameeullah, Hiffza Qadeer Abbasi, Zainab Masood, Tijen Demiral Sert, Noreen Aslam, Turgay Pekdemir, Mustafa Imren, Vahdettin Çiftçi, Kiran Saba, Muhammad Suleman Malik, Fatima Ijaz, Neelam Batool, Bushra Mirza, Mohammad Tahir Waheed

**Affiliations:** ^1^ Department of Biology, University of Haripur, Haripur, Pakistan; ^2^ Department of Field Crops, Faculty of Agriculture, Bolu Abant Izzet Baysal University, Bolu, Türkiye; ^3^ Centre for Innovative Food Technologies Development, Application and Research, Bolu Abant Izzet Baysal University, Bolu, Türkiye; ^4^ Department of Biochemistry, Quaid-i-Azam University, Islamabad, Pakistan; ^5^ Department of Biology, Faculty of Engineering and Natural Sciences, Süleyman Demirel University, Isparta, Türkiye; ^6^ Department of Biology, Faculty of Science and Literature, Bolu Abant Izzet Baysal University, Bolu, Türkiye; ^7^ Department of Chemical Engineering, Faculty of Engineering, Bolu Abant Izzet Baysal University, Bolu, Türkiye; ^8^ Department of Plant Protection, Faculty of Agriculture, Bolu Abant Izzet Baysal University, Bolu, Türkiye; ^9^ Department of Biochemistry, Faculty of Life Sciences, Shaheed Benazir Bhutto Women University, Peshawar, Pakistan

**Keywords:** *Brassica oleracea*, antidiabetic, alloxan, antioxidant enzymes, lipid peroxidation, histopathology, flavonoids, ABC transporter

## Abstract

**Background:**

The global prevalence of diabetes among adults over 18 years of age is expected to increase from 10.5% to 12.2% (between 2021 and 2045). Plants can be a cost-effective source of flavonoids like quercetin and kaempferol with anti-diabetic properties.

**Methodology:**

We aimed to assess the antidiabetic potential of leaves of *Brassica oleracea* cvs. Green Sprout and Marathon. Further, flavonoid contents were measured in broccoli leaves grown under light and dark conditions. The methanolic extracts of Green Sprout (GSL-M) and Marathon (ML-M) were first evaluated *in vitro* for their α-amylase and α-glucosidase inhibitory potential and then for antidiabetic activity *in vivo* in alloxan-induced diabetic rat models.

**Results:**

Treatment with plant extracts promoted the reduced glutathione (GSH) content and CAT, POD, and SOD activities in the pancreas, liver, kidney, heart, and brain of diabetic rats, whereas lowered lipid peroxidation, H_2_O_2_, and nitrite concentrations. The histopathological studies revealed the protective effect of plant extracts at high dose (300 mg/kg), which could be due to broccoli’s rich content of chlorogenic acid, quercetin, and kaempferol. Strikingly, etiolated leaves of broccoli manifested higher levels of quercetin and kaempferol than green ones. The putative role of an ABC transporter in the accumulation of quercetin and kaempferol in etiolated leaves was observed as evaluated by qRT-PCR and *in silico* analyses.

**Conclusion:**

In conclusion, the present study shows a strong link between the antidiabetic potential of broccoli due to the presence of chlorogenic acid, quercetin, and kaempferol and the role of an ABC transporter in their accumulation within the vacuole.

## 1 Introduction

Diabetes mellitus is a chronic illness characterized by severe metabolic disturbances. According to the statistics published by the International Diabetes Federation (IDF), around 537 million people suffered from diabetes in 2021, and this number is expected to rise to 783 million by 2045 (Sun et al., 2022). Complications of diabetes are generally categorized into microvascular (neuro, nephron, and retinopathy) and macrovascular (coronary heart disease, stroke, and peripheral vascular disease). While taking into account the intensity of these complications, this division is further elaborated as short-term or metabolic acute complications such as hypoglycemia and ketoacidosis or systemic late complications such as chronic infections like diabetic foot disease, diabetic nephropathy, and diabetic neuropathy (Ullah et al., 2016).

Oxidative stress is a pathological state caused by either excessive production of reactive oxygen species (ROS) and reactive nitrogen species (RNS) or inadequate removal from the cells. The production of free radicals is directly involved in the tissue damage and initiation of the pro-inflammatory cascade. Several experimental and clinical studies have shown direct evidence of the involvement of oxidative stress in the pathogenesis and progression of hyperglycemia-related vascular complications (Thakur et al., 2017). Antioxidants are endogenous or exogenous entities that prevent cellular damage by inhibiting the production of free radicals or by scavenging the free radicals from the cell (Shahrajabian and Sun, 2023). Significant considered biomarkers for investigating the effect of antioxidants in diabetic animal models are superoxide dismutase (SOD), catalase (CAT), peroxidase (POD), and glutathione (GSH) (Haan and Cooper, 2011). Similarly, lipid peroxidation is the most emphasized domain of research on oxidative stress that contributes to the production of ROS through a series of intermediates Valgimigli (2023).

Over the years, phytotherapy has become a significant source for managing diabetes mellitus. Despite the vast amount of data on the traditional use of plants for treating diabetes, most of the used plant species are neglected due to insufficient clinical evidence or poor quality of clinical trials (Bagherniya et al., 2018). In recent years, many plants have been thoroughly studied for their antidiabetic potential while considering their phytochemistry and their underlying mechanism of action in detail, such as onion (*Allium cepa* L.), neem (*Azadirachta indica* A. Juss.), basil (*Ocimum tenuiflorum* L.) and fenugreek (*Trigonella foenum-graecum* L.) (Governa et al., 2018).

Broccoli, a well-known medicinal plant, scientifically known as *Brassica oleracea* var*.* italica is a member of the family Brassicaceae. This cruciferous vegetable is known to have a large amount of medicinally active phytochemicals, including phenols and flavonoids (Ravikumar, 2015). Chlorogenic acid and Flavonols such as quercetin and kaempferol are the major metabolites found in broccoli (Vollmannová et al., 2024). Kaempferol exerts its antidiabetic effects by enhancing the expression and activation of AMP-activated proteins, thereby promoting cellular energy metabolism. Additionally, it reduces cellular apoptosis by suppressing caspase-3 activity and stimulates insulin secretion from pancreatic beta cells (Zhang et al., 2011). Quercetin exerts a hypoglycemic effect in animal models (Sok Yen et al., 2021) as well as reduces intestinal glucose uptake and decreases postprandial blood glucose levels in diabetic mice through the inhibition of the GLUT2 glucose transporter (Alkhalidy et al., 2018). *In vivo* studies with animals and clinical trials to assess the antidiabetic activity of chlorogenic acid-rich foods and supplements or pure chlorogenic acid showed that chlorogenic acid has antioxidant, anti-inflammatory, anti-obesity, anti-dyslipidemia, antidiabetic, and antihypertensive properties, which can serve for the prevention and treatment of metabolic syndrome and associated disorders (Santana-Gálvez et al., 2017). Thus, the presence of these metabolites in broccoli is worthwhile having anti-diabetic and anti-obese functional food aside from its chemo-protective and cardio-protective roles (Syed et al., 2023).

Flavonoids have diverse roles in plant development, physiology, and ecology and are primarily stored in vacuoles. Various transporters have been reported for their vacuolar transport activity of flavonoids in addition to anthocyanins. In *Arabidopsis*, AtBCC2 manifested the vacuolar transport of anthocyanins but also for flavones luteolin 7-O-glucoside (L7G) and apigenin 7-O-glucoside (A7G) and flavonols kaempferol 3-O-glucoside (K3G) and quercetin 3-O-glucoside (Q3G). MtMATE2 could uptake kaempferol 7-O-glucoside (K7G) and its product kaempferol 7-O-glucoside malonate (K7GM) when using heterologous expression system of yeast vacuolar vesicles, although exhibited higher transport rate for anthocyanins (Zhao et al., 2011). A plasma membrane-localized MATE-type transporter (LaMATE2) of white lupin could transport genistein but no other flavonoids, including kaempferol, in a yeast expression system (Biała-Leonhard et al., 2021). A growing body of research shows that MATE and ABC transporter gene families are mainly involved in flavonoid transport in flowering plants (Martinoia, 2017).

The present study aimed to explore the antidiabetic potential of two cultivars of *Brassica oleracea* var. italica, the Green Sprout, and Marathon leaves’ extracts at two different concentrations, i.e., 150 mg/kg b. w and 300 mg/kg b. w, studying their antioxidant effect on five vital organs in diabetic rat models (the dose concentration was selected based on a previous study done by Lutfiyati et al. (2018), as well as the role of a putative flavonoid transporter protein and its involvement in the accumulation of flavonoids including quercetin and kaempferol in leaves of broccoli seedlings. The relatively high concentrations of broccoli extracts were selected for testing because they were the crude extracts of selected cultivars. Plant leaves were chosen because of their high biomass and ease of processing and because they are plants’ bio-factories containing many essential components. In this study, we selected commercial methanol as a solvent for extraction as methanol extracts of different plant leaves have previously been reported to show the maximum concentration of flavonoids and phenols due to polarity (Dhawan and Gupta, 2017). We also measured the levels of chlorogenic acid and flavonoids, including quercetin and kaempferol, in the leaves of broccoli under different physiological conditions to evaluate further the probable involvement of a putative transporter in the accumulation of these flavonoids in the vacuole.

## 2 Materials and methods

### 2.1 Plant collection, extract preparation, physiological and molecular analyses

For the bioassays, commercially available seeds of two broccoli cultivars, i.e., Green Sprout and Marathon, were used, and plants were grown under controlled conditions. Broccoli was grown in the peat medium from Klassman TS 1 including micronutrients as well as potassium (mg K2O/l): 180, magnesium (mg Mg/L): 100, phosphorus (mg P2O5/l): 160, and nitrogen (mg N/L): 140. The details can be found at https://agrohoum.gr/datafiles/files/TS1%20FINE.pdf (accessed on August 15, 2024). These seedlings were maintained under controlled conditions in a plant growth room with a 65% humidity level, 25°C temperature, a 16-h light/eight-hour dark cycle photoperiod, and light intensity of about 250 µmol m2s-1. Plants were watered every 5 days. Since environmental conditions like soil, humidity, temperature, and light conditions are crucial (Li et al., 2020), the conditions were maintained stringently throughout the growing period of the broccoli plants. After 2 months of germination, leaves from the full-grown plants were collected, washed with distilled water, and allowed to shade dry. After complete drying, leaves were ground to fine powder. Eighty grams of finely grounded leaves of each cultivar were weighed and soaked in commercial-grade methanol for 2 days with occasional shaking, and the filtrate was separated using Whatmann’s no. 1 filter paper.

For the analyses of real-time qPCR and HPLC-based quantification of quercetin and kaempferol contents, the 7-day-old seedlings of Green Sprout cultivar were exposed to dark periods for 7 days but for 2 weeks for chlorophyll content measurement (Zu et al., 2006; Saba et al., 2020; Aslam et al., 2022). The dark condition denotes the continuous exposure to dark from day 7^th^ to day 15^th^ of the broccoli growing stage, and the light condition refers to the 16 h light/8 h dark cycle during the growth period. The chlorophyll contents of etiolated and green seedlings were measured according to Sameeullah et al. (2021).

### 2.2 *In vitro* antidiabetic activity

*In vitro,* the antidiabetic activity of both Broccoli cultivars was determined by the following two assays.

#### 2.2.1 α-glucosidase inhibition assay

α-glucosidase inhibition activity of methanol extract of both cultivars was determined by the protocol of Jabeen et al. (2014). 100 μL of Acarbose, the standard (20 mg/mL) was added to 50 μL α-glucosidase that was prepared at the concentration of 1 U/mL in 0.1 M Phosphate buffer (PH 6.9). pre-incubation at 37°C for 20 min was done. After preincubation 10 μL of 10 mM P-nitrophenyl-α-D-glucopyranoside (the substrate) was added and the mixture was incubated at 30°C for 30 min 650 μL of 1 M sodium bicarbonate was used to stop the reactions The activity of extracts was measured by preparing three serial dilutions, having the concentrations 1000 μg/mL, 500 μg/mL, and 250 μg/mL to calculate the dose-dependent effect of the plant extract. Absorbance was measured by using spectrophotometer at 405 nm. Enzyme activity was calculated as percentage inhibition by using the following formula

Percentage inhibition=A405 of control−A405 of tratmentA405 of control]×100

#### 2.2.2 α-amylase inhibition assay

α-amylase inhibition assay was performed to verify the antidiabetic potential of the methanol extract of Green Sprout and Marathon cultivars, according to the method of Keerthana et al. (2013). The stock solutions of the acarbose as well as test samples were prepared in water. 100 μL of acarbose (prepared as serial dilutions of concentrations 2–20 mg/mL was added to 100 μL of α-amylase (1 U/mL) and 200 μL of sodium phosphate buffer (20 mM, pH 6.9) to get 0.5–5.0 mg/mL final working concentration. Pre-incubation was done at 25°C for 10 min, and 200 μL of 1% starch (the substrate) prepared in 20 mM sodium phosphate buffer (pH 6.9) was added. The reaction mixtures were incubated at 25°C for 10 min. The reactions were stopped by incubating the mixture in a boiling water bath for 5 min after adding 1 mL of dinitrosalicylic acid. The reaction mixtures were cooled to room temperature, diluted to 1:5 ratio with water, and absorbance was measured in a spectrophotometer (at 540 nm. The extracts were checked for activity at three different concentrations: 1000 μg/mL, 500 μg/mL, and 250 μg/mL to estimate the dose dependent α-amylase inhibition potential of plant extracts. The percentage of inhibition of enzyme activity was calculated as

Percentage inhibition=A405 of control−A405 of tratmentA405 of control]×100

### 2.3 Animal trials

#### 2.3.1 Animals

Twenty-one adult male Sprauge-Dewaly rats weighing 150–200 g each were used in the present study that were kept and bred at the primate facility, Quaid-i-Azam University, Islamabad, Pakistan (under permission letter#: BEC-FBS-QAU2019-164 from Bioethical Committee). Only male rats were used in this experiment to avoid fluctuations in the readings due to hormonal changes that happened in the female rats because of their estrous cycle. Animals were kept in cages at room temperature (25°C ± 30°C), provided with an adequate amount of food and water, and a standard light-dark cycle (12 light +12 h dark)—experimental protocol as approved by the ethical board committee of Quaid-i-Azam University. Details of experimental groups are given below in Table 1.

**TABLE 1:** Administration of control and treatment groups of rats.

Group	Characteristics	Treatment
Group I	Normal animals	Normal diet + vehicle control (10% DMSO) (n = 3)
Group II	Diabetic Negative Control	Normal diet + vehicle control (10% DMSO) (n = 3)
Group III	Diabetic Positive control	Normal diet + Glibenclamide (10 mg/kg) (n = 3)
Group IV	Diabetic + ML-M Low dose	Normal diet + Marathon leaves methanol extract low dose (150 mg/kg b.w in 10% DMSO)
Group V	Diabetic + ML-M high dose	Normal diet + Marathon leaves methanol extract high dose (300 mg/kg b.w in 10% DMSO)
Group VI	Diabetic + GSL-M low dose	Normal diet + Green Sprout leaves methanol extract low dose (150 mg/kg b.w in 10% DMSO)
Group VII	Diabetic + GSL-M high dose	Normal diet + Green Sprout leaves methanol extract high dose (300 mg/kg b.w in 10% DMSO)

#### 2.3.2 Diabetes induction and multiple-dose studies in rats

For the induction of diabetes, 120 mg/kg alloxan monohydrate (Sigma-Aldrich, cat# A7413) solution was injected intraperitoneally into each group of rats except the normal control group. All the treatments were administered orally. Animals were fed with 70% glucose solution to overcome post-alloxan hypoglycemia. After 24 h, the blood glucose level of each rat was checked with a glucometer (Accu-Chek active blood glucose meter). Animals with blood glucose concentrations ≥200 mg/dL were considered diabetic and were preceded for multiple-dose studies. Excluding groups I and II, all other groups were given their respective doses for seven alternate days, with blood glucose concentration measured each day.

#### 2.3.3 Dissection

After 14 days of dosing, animals were anesthetized and sacrificed for organ collection. Five vital organs pancreas, liver, heart, brain, and kidney, were collected from each rat. Half of each organ was preserved in normal saline (0.5% NaCl) solution for biochemical analysis, and the other half in 10% formalin solution for histology.

### 2.4 Biochemical analysis

#### 2.4.1 Tissue homogenate preparation

100 mg of each organ (pancreas, liver, heart, brain, and kidney) was taken and homogenized in potassium phosphate buffer (1 mM potassium phosphate +1 mM EDTA, pH 7.4). Homogenate was centrifuged at 10,000 rpm while maintaining the temperature at 4°C for 30 min. The supernatant was separated, stored at −20°C, and used for the enzymatic and non-enzymatic antioxidant profiling.

#### 2.4.2 Enzymatic antioxidant profiling

Activities of enzymatic oxidants, i.e., catalase (CAT), peroxidase (POD), and superoxide dismutase (SOD), were checked for each separated organ from all experimental groups. CAT and POD activities were assayed according to Maehly’s protocol (Maehly, 1954). In the assessment of catalase activity, the reacting solution consisted of 625 μL of 50 mM of potassium phosphate buffer (pH 5), 100 μL of 5.9 mM H_2_O_2_, and 35 μL enzyme extract. After a minute, changes in absorbance at 240 nm were measured. One unit of catalase activity was stated as an absorbance change of 0.01 units/min. Whereas in the case of assessment of peroxidase levels, the reaction solution contained 40 mM hydrogen peroxide (75 μL), 20 mM guaiacol (25 μL), and 625 μL of 50 mM potassium phosphate buffer (pH 5.0), and 25 μL of tissue homogenate. After 1 min, the change in absorbance was measured at 470 nm. One unit POD activity is considered as change in absorbance of 0.01 as units/min. The activity of SOD in tissue homogenates was assessed by following the method of Kakkar et al. (1984). Assessment of SOD activity was done by the reaction of phenazinemethosulphate and sodium pyrophosphate buffer. Tissue homogenate was centrifuged at 1,500 × g for 10 min and then at 10,000 × g for 15 min. The supernatant was heaped and 150 μL of it was added to the aliquot containing 600 μL of 0.052 mM sodium pyrophosphate buffer (pH 7.0) and 186 mM of phenazinemethosulphate (50 μL). To initiate the enzymatic reaction, 100 μL of 780 μM NADH was added. After 1 min, glacial acetic acid (500 μL) was added to the reaction mixture to stop the reaction. Optical density was measured at 540 nm to recapitulate the color intensity. Results were evaluated in units/mg protein.

#### 2.4.3 Non-enzymatic antioxidant profiling

In non-enzymatic profiling, reduced glutathione (GSH) level was measured. Hydrogen peroxide (H_2_O_2_), tissue nitrite levels, and thiobarbituric acid reactive substances (TBARS) levels were assessed as oxidative stress markers. Levels of GSH in all organs were quantified using the method of Jollow et al. (1974). 4% sulfosalicylic acid (500 μL) was added to the tissue homogenate (500 μL), and precipitation occurred. After 1 h of incubation at 4°C the samples were centrifuged for 20 min at 1,200 × g. 33 μL supernatant was collected and mixed into aliquots consisting of 900 μL of 0.1 M potassium phosphate buffer (pH 7.4) and 66 μL of 100 mM DTNB. As a result of this reaction between GSH and DNTB a yellow-colored complex of reduced glutathione was produced. Absorbance was measured at 405 nm through micro microplate reader. The GSH activity was measured as μM GSH/g tissue Hydrogen peroxide levels were assessed using Pick and Keisari’s protocol (Pick and Keisari, 1981). The H_2_O_2_ horseradish peroxidase enzyme engenders the oxidation of phenol red. reaction mixture contained 500 μL of 0.05 M phosphate buffer (pH 7), 100 μL of homogenate was added in conjunction with 100 μL of 0.28 nM phenol red solution, 250 μL of 5.5 nM dextrose and horse radish peroxidase (8.5 units). The reaction mixture was left at room temperature for 60 min 100 μL of NaOH (10 N) was then added to stop the reaction. Then mixture tubes were centrifuged for 5–10 min at 800 × g using a spectrophotometer the absorbance of the collected supernatant was recorded against the reagent as a blank at 559 nm. Production of H_2_O_2_ was assessed as nM H_2_O_2_/min/mg tissue based on the standard curve of H_2_O_2_ oxidized phenol red. The status of tissue nitrites was measured using a standard sodium nitrite curve (Grisham et al., 1996). For the deproteinization of tissue samples (100 mg each) equal quantity, i.e. 100 μL each of both 5% ZnSO4 and 0.3 M NaOH was used. The mixture was centrifuged at 6,400 × g for 15–20 min later 20 μL supernatant was mixed with Griess reagent (1.0 mL) in the cuvette and at 570 nm, color change was estimated by measuring the absorbance. 1 mL Griess reagent was used as a blank. The standard curve of sodium nitrite was used for the evaluation of nitrite concentration in living tissues. The extent of lipid peroxidation (TBARS levels) was measured by the protocol of Iqbal et al. (1996). The reaction mixture contained 0.1 M phosphate buffer of 290 μL (pH 7.4), 100 mM ferric chloride (10 μL), 100 mM ascorbic acid (100 μL), and 100 μL of homogenized tissue sample. The reaction mixture was incubated at 37°C for about 1 h in the shaking water bath. 10% trichloroacetic acid (500 μL) was added to the above reaction mixture to inhibit the reaction. Then 0.67% thiobarbituric acid (500 μL) was added, and the reaction tubes were kept in the water bath for 20 min. After that, the tubes were removed from the water bath placed in the crushed ice bath for 5 min, and were centrifuged at 2500 × g for 12–15 min. Absorbance was calculated at 540 nm against a blank having reagent using a microplate reader. By using the molar extinction coefficient of 1.56 × 105/M/cm, results were recorded as nM of TBARS formed per min per mg tissue at 37°C.

### 2.5 Histopathological examination

Histopathological assessment was done via a paraffin-embedded staining procedure (Hristu et al., 2021). The fresh tissues of all isolated organs were sliced into small pieces and fixed in 10% formalin. The tissues were then secured on hard solid blocks via paraffin embedding. Slides were prepared by sectioning 3–4 µm thin layers of the embedded tissue samples and staining with hematoxylin and eosin. Afterward, the slides were examined under the light microscope (DIALUX 20EB) at 10X and photographed via an HDCE-50B camera.

### 2.6 *In silico* analysis of a putative ABC transporter

The full-length protein sequence of the putative ABC transporter (BolC8t51326H), possibly responsible for the accumulation of quercetin and kaempferol in broccoli, was retrieved from the Brassicaceae Database (BRAD) (Chen et al., 2021). The phylogenetic tree was constructed using the neighbor-joining method using MATE, ABC, and GST gene family members responsible for flavonoid transport in different plant species. The membrane topology of the putative transporter was drawn using the online resource Protter (Omasits et al., 2013). The subcellular localization of the ABC transporter was predicted using LocTree3 (Goldberg et al., 2014).

### 2.7 Real-time qPCR analysis of ABC transporter in leaves of broccoli seedlings

After *in silico* analysis of a putative ABC transporter (BolC8t51326H) and its predicted localization, its expression levels were further analyzed by a real-time qPCR to explore its presumed role in the accumulation of flavonoids like quercetin and kaempferol in broccoli leaves. RNA isolation, cDNA synthesis, and real-time PCR were performed following the procedures reported earlier (Sameeullah et al., 2013; Sameeullah et al., 2021). The gene-specific primer set for quantitative real-time PCR was BolC8t51326H-F 5′-CTC​TCG​GTG​AGT​GAA​AAG​CTG-3′ and BolC8t51326H-R 5′-GTC​TGC​GAA​TCA​ACA​GAC​GC-3'. As reported earlier, *ACT2* was used as a reference gene (Ren et al., 2022).

### 2.8 Detection of phenolic substances in broccoli leaves by HPLC

Chromatographic analysis of chlorogenic acid, quercetin, and kaempferol was done according to Zu et al. (2006). 7-day-old broccoli seedlings were exposed to light (control) and dark conditions (etiolated) for 7 days. Then, the fresh green or etiolated leaves were cleaned and dried at 60°C. 2 g of powdered leaves were extracted with 30 mL of 85% ethanol under 80 kHz, 45°C in an ultrasonic extraction device for 30 min, repeated twice. The extract was filtered, and the filtrate was dried at 50°C under reduced pressure in a rotary evaporator. The dried extract was dissolved in the mobile phase methanol. The extract was injected directly after filtering through a filter paper and a 0.45 mm membrane filter (Millipore).

Chromatographic analysis was performed by Shimadzu HPLC SPD-M 10A vp diode array detection (DAD) detector and Agilent Eclipse XDB-C18 (250 × 4.60 mm) 5-micron column. The mobile phase was A: 3% acetic acid, B: Methanol. Flow rate and injection volume were 0.8 mL/min and 20 μL, respectively. Chlorogenic acid, quercetin, and kaempferol were quantified by DAD following HPLC separation at 278 nm. The chromatographic peaks of the analytes were confirmed by comparing their retention time and UV spectra with those of the reference standards. Quantification was performed by integrating the peaks using the external standard method. All chromatographic operations were performed at room temperature.

### 2.9 Statistical analysis

Graph pad Prism version 5 and Statistica 8.1 software were used to analyze the data statistically. Data was compared to the respective control groups by one-way ANOVA followed by Tukey’s comparison and two-way ANOVA followed by Bonferroni post-analysis. *P*-value ≤ 0.05 was considered statistically significant.

## 3 Results and discussion

### 3.1 Effect of ML-M and GSL-M on inhibition of α-glucosidase and α-amylase enzymes

ML-M (Marathon methanolic extract) and GSL-M (green sprout methanolic extract) showed a very promising effect in inhibiting α-glucosidase and α-amylase enzymes in a concentration-dependent manner, where ML-M showed the highest percentage inhibition equivalent to 69.2% ± 3.6% at the concentration of 1000 μg/mL in case of α-glucosidase inhibition assay. At the same time, GSL-M showed 52.3% ± 2.5% inhibition at the same concentration. Acarbose was used as a positive control in this assay, showing 90.6% ± 1.1% inhibition at 1000 μg/mL concentration. Results are summarized in Table 2 α-glucosidase inhibitors delay the absorption of carbohydrates in the small intestine, thus reducing the postprandial blood glucose levels (Mohd Bukhari et al., 2017). A similar mode of action could be considered for ML-M and GSL-M extracts.

**TABLE 2:** α-glucosidase and α-amylase inhibition activity of ML-M and GSL-M.

Concentration µg/mL	Percentage inhibition					
	α-glucosidase enzyme			α-amylase enzyme		
	Acarbose	ML-M	GSL-M	Acarbose	ML-M	GSL-M
1000 μg/mL	90.6 ± 1.1	69.2 ± 3.6^*^	52.3 ± 2.5^**^	91.6 ± 1.1	64.11 ± 3.6	58.3 ± 2.5^*^
500 μg/mL	85.3 ± 1.5	57.0 ± 3.4	27.4 ± 1.0^**^	83.3 ± 1.5	55.5 ± 3.4	44.4 ± 1.0^**^
250 μg/mL	78.9 ± 1.6	53.3 ± 2.7	20.4 ± 2.1^**^	72.2 ± 1.6	36.1 ± 2.7^**^	33.3 ± 2.1^**^
IC50µg/mL	35.4	213.1	2,887	97.65	451	649.3

Values are presented as mean ± SD (n = 3). Acarbose was used as a positive control. All groups were compared with positive control Acarbose at respective concentrations. Mean values with superscripts (*) are significantly different at *P ≤ 0.05 and **P ≤ 0.01.

While checking the α-amylase inhibition activity of ML-M and GSL-M, ML-M showed 64.11% ± 3.6% inhibition at the concentration of 1000 μg/mL, GSL-M showed 58.3% ± 2.5% inhibition at the highest concentration. In this assay, acarbose, the positive control, showed a 91.6% ± 1.1% inhibition at 1000 μg/mL concentration. Results are displayed in Table 2 α-amylase inhibitors or “starch blockers” prevent the hydrolysis of starch and other oligosaccharides into simple sugars (Wickramaratne et al., 2016). The prominent activity of ML-M in inhibiting the α-amylase could be due to the presence of polar phyto-constituents.

### 3.2 Effect of ML-M and GSL-M on plasma glucose levels (mg/dL) in diabetic animals

**FIGURE 1:**
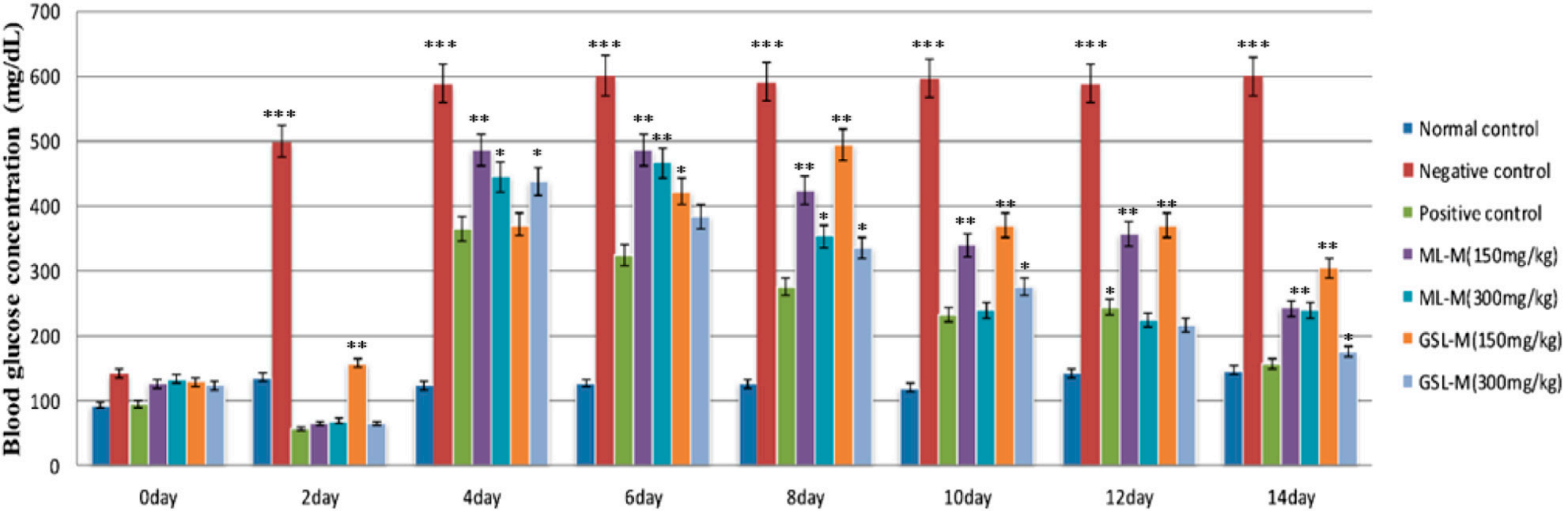
Blood glucose concentration (mg/dL) at different intervals in experimental groups. Normal control: Non-diabetic healthy animals. Negative control: Untreated diabetic animals. Positive control: Glibenclamide-treated diabetic animals. ML-M: Marathon leaves methanol extract treated diabetic animals. GSL-M: Green sprout leaves methanol extract treated diabetic animals. All groups were compared to the normal control group using two-way ANOVA followed by Bonferroni’s test. *P ≤ 0.05, **P ≤ 0.01, ***P ≤ 0.001.

ML-M and GSL-M showed a very promising effect as both extracts significantly (*p* ≤ 0.05) lowered the blood glucose concentration in alloxan-induced diabetic animals in a dose-dependent manner. After recovering from the initial hypoglycemic state generated by the alloxan injection, blood glucose concentration was significantly high in negative diabetic control (588 ± 2.1 mg/dL), which remained consistently high throughout the experiment. The positive control, i.e., Glibenclamide, restored the elevated blood glucose level from 365 ± 1.3 mg/dL to 156 ± 1.16 mg/dL on day 14 before dissection, slightly higher than normal animals. While observing the plant-treated groups, GSL-M at its high concentration (300 mg/kg) appeared to be the most effective extract (decreasing the blood glucose concentration from 437 ± 2.61 mg/dL to 176 ± 1 mg/dL). ML-M at its high dose (300 mg/kg) also showed significant hypoglycemic activity in a time-dependent manner (decreasing the blood glucose concentration from 485 ± 2 mg/dL to 240 ± 1.16 mg/dL). The plant-treated groups showed comparable effects to that of the standard drug Glibenclamide. This hypoglycemic activity of broccoli extracts might be due to the presence of kaempferol and quercetin, which regulate blood glucose levels by inhibiting the activity of glucose transporters and *α*-glucosidase, reducing hepatic glucose production (Sok Yen et al., 2021; Yang et al., 2022). It also prevents glucose intolerance in diet-challenged animal models (Ravikumar, 2015). The effect of ML-M and GSL-M on plasma glucose levels in diabetic animals is shown in Figure 1. In this assay, the blood glucose level of all diabetic groups was compared with normal, non-diabetic healthy animals.

### 3.3 Effect of ML-M and GSL-M on the status of antioxidants (CAT, POD, SOD, GSH)

**FIGURE 2:**
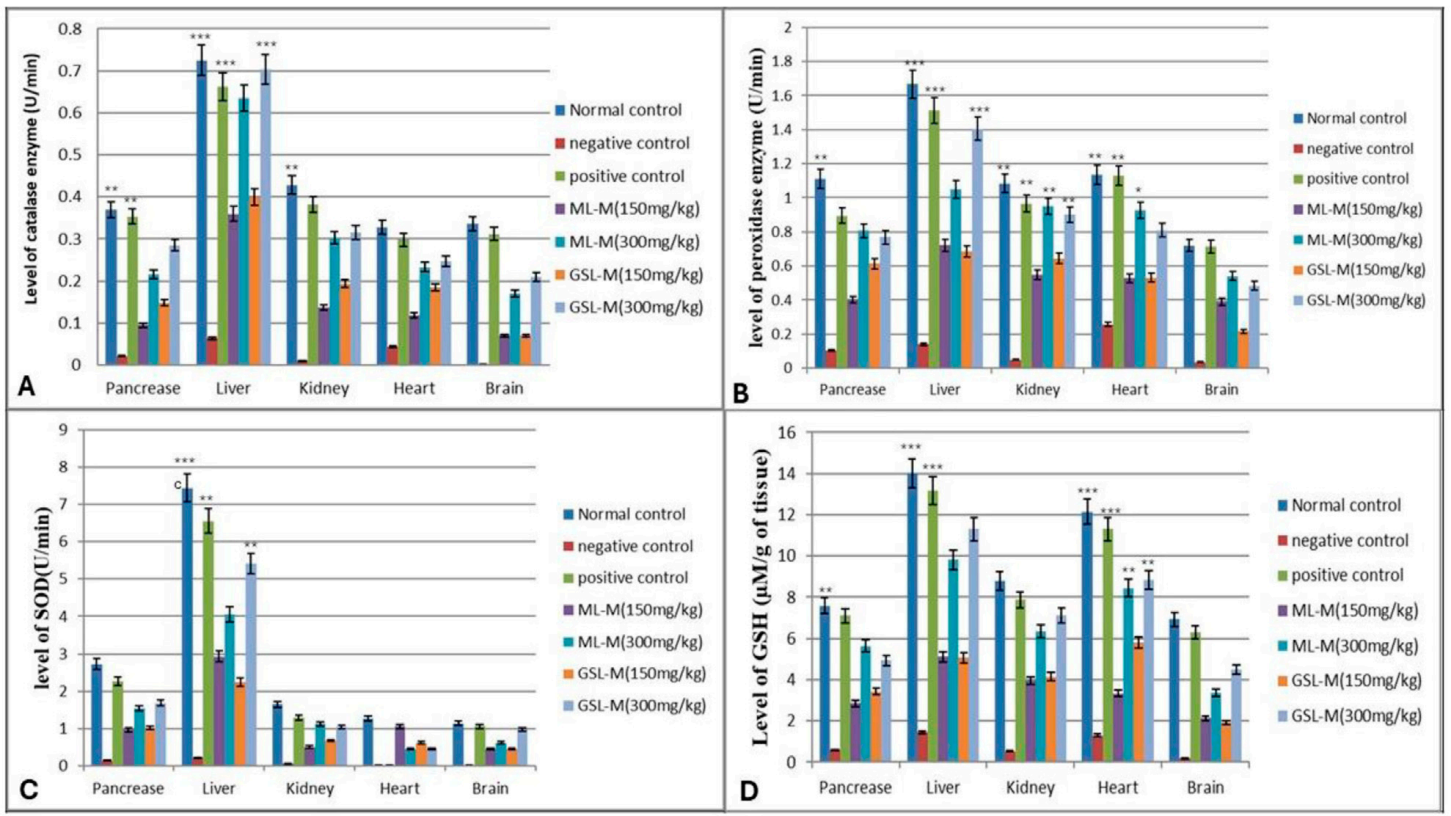
Effect of 150 mg/kg and 300 mg/kg doses of ML-M and GSL-M methanolic extracts on the status of antioxidants in the pancreas, liver, kidney, heart, and brain tissues of diabetic animals. **(A)** Catalase (CAT) activity (U/min). **(B)** Peroxidase (POD) activity (U/min). **(C)** Superoxide dismutase (SOD) activity (U/min). **(D)** Reduced glutathione (GSH) level (µM/g of tissue). Normal control: Non-diabetic healthy animals. Negative control: Untreated diabetic animals. Positive control: Glibenclamide-treated diabetic animals. ML-M: Marathon leaves methanol extract treated diabetic animals. GSL-M: Green sprout leaves methanol extract treated diabetic animals. All groups were compared to negative control groups using one-way ANOVA followed by Tukey’s comparison. *P ≤ 0.05, **P ≤ 0.01, ***P ≤ 0.001.

The level of a nonenzymatic antioxidant, reduced glutathione (GSH), and activities of antioxidant enzymes (CAT, POD, and SOD) were studied in the pancreas, liver, kidney, heart, and brain tissues of all experimental groups, and enzyme activity was measured in Unit/minute. Induction of diabetes by alloxan injection remarkably reduced the activities of all antioxidant enzymes studied, i.e., CAT, POD, and SOD, as well as the GSH level. Glibenclamide (10 mg/kg b. w) showed a positive impact and reversed back the effect of alloxan in all organs by regulating CAT enzyme activities (pancreas 0.3535 ± 0.16 U/min; liver 0.6616 ± 0.02 U/min; kidney 0.3819 ± 0.001 U/min; heart 0.298 ± 0.024 U/min; brain 0.3119 ± 0.001 U/min). We observed that the animals treated with ML-M and GSL-M showed improved antioxidant enzyme status much closer to that of standard drugs than that of the untreated diabetic group. The level of CAT drastically dropped down (*p* ≤ 0.05) in all organs of the untreated diabetic group (pancreas 0.0214 ± 0.1 U/min; liver 0.0634 ± 0.01 U/min; kidney 0.010 ± 0.001 U/min; heart 0.0433 ± 0.01 U/min; brain 0.00098 ± 0.0001 U/min). Treatment with ML-M and GSL-M high dose (300 mg/kg) significantly reversed the dropped levels of CAT near the normal healthy group (Figure 2A).

The status of the other protective antioxidant enzyme, POD, in the ML-M and GSL-M-treated diabetic rats, was compared to its level in the organs of untreated diabetic control and standard drug-treated positive control. The drastic decrease in the level of peroxidase activity in all organs of untreated diabetic control showed oxidative stress due to an altered balance between the production of free radicals and antioxidant enzyme activities. Compared to the diabetic control, glibenclamide showed a protective effect by elevating the activity of POD in all tested organs, especially in the liver, where the POD activity was measured as 1.513 ± 0.31 U/min. Among the plant extract-treated groups, ML-M, at its high concentration, significantly enhanced the POD activity in all cell types. The most prominent effect was shown in the liver cells, where the activity of POD was noted as 0.1402 ± 0.1 U/min in the negative control but was increased up to 1.0946 ± 0.5 U/min in the ML-M (300 mg/kg) treated group. GSL-M also showed a protective effect regarding the activity level of POD in a dose-dependent manner, where a high dose proved more effective than a low dose (Figure 2B).

Similar results were obtained in the case of SOD activity and GSH level. Glibenclamide was used as a positive control and significantly enhanced SOD activity and GSH level, recorded as 6.556 ± 0.37 U/min (Figure 2C) and 13.17 ± 1.12 µM (Figure 2D), respectively. Regarding SOD activity in the plant extract-treated groups, the most effective extract was ML-M at its high concentration. The prominent difference was observed in the hepatic cells, where the activity of SOD was 0.2126 ± 0.1 U/min in the negative control, whereas in the ML-M treated group, it was increased up to 4.0476 ± 0.5 U/min. Trends in the GSH content were similar to other enzyme activities, where the levels of GSH were significantly improved in GSL-M and ML-M treated groups compared to the non-treated diabetic group (negative control).

Oxidative stress is the main culprit behind cellular damage in patients with diabetes mellitus. Against this oxidative stress-induced damage in the cells, antioxidant enzymes such as CAT, POD, and SOD present the first line of defense at the cellular level. SOD catalyzes the conversion of superoxide into hydrogen peroxide (Halliwell, 2006). CAT, a haemo-protein, scavenges the hydrogen peroxide from the cell to protect against tissue damage caused by oxidative stress (Sahreen et al., 2014). The reduced form of glutathione (GSH) plays a vital role as a scavenger of electrophilic and oxidant species either directly or through enzymatic catalysis to maintain cell homeostasis and glutathione peroxidase scavenges H_2_O_2_ to water by using GSH as a hydrogen donor (Maritim et al., 2003). Our study has suggested the protective potential of the two cultivars of *Brassica oleracea* var. italica against alloxan-induced oxidative stress in diabetic animals, as it significantly enhanced the activities of essential antioxidant enzymes and GSH at the cellular level in all vital organs.

### 3.4 Effect of ML-M and GSL-M on the status of oxidative stress markers

**FIGURE 3:**
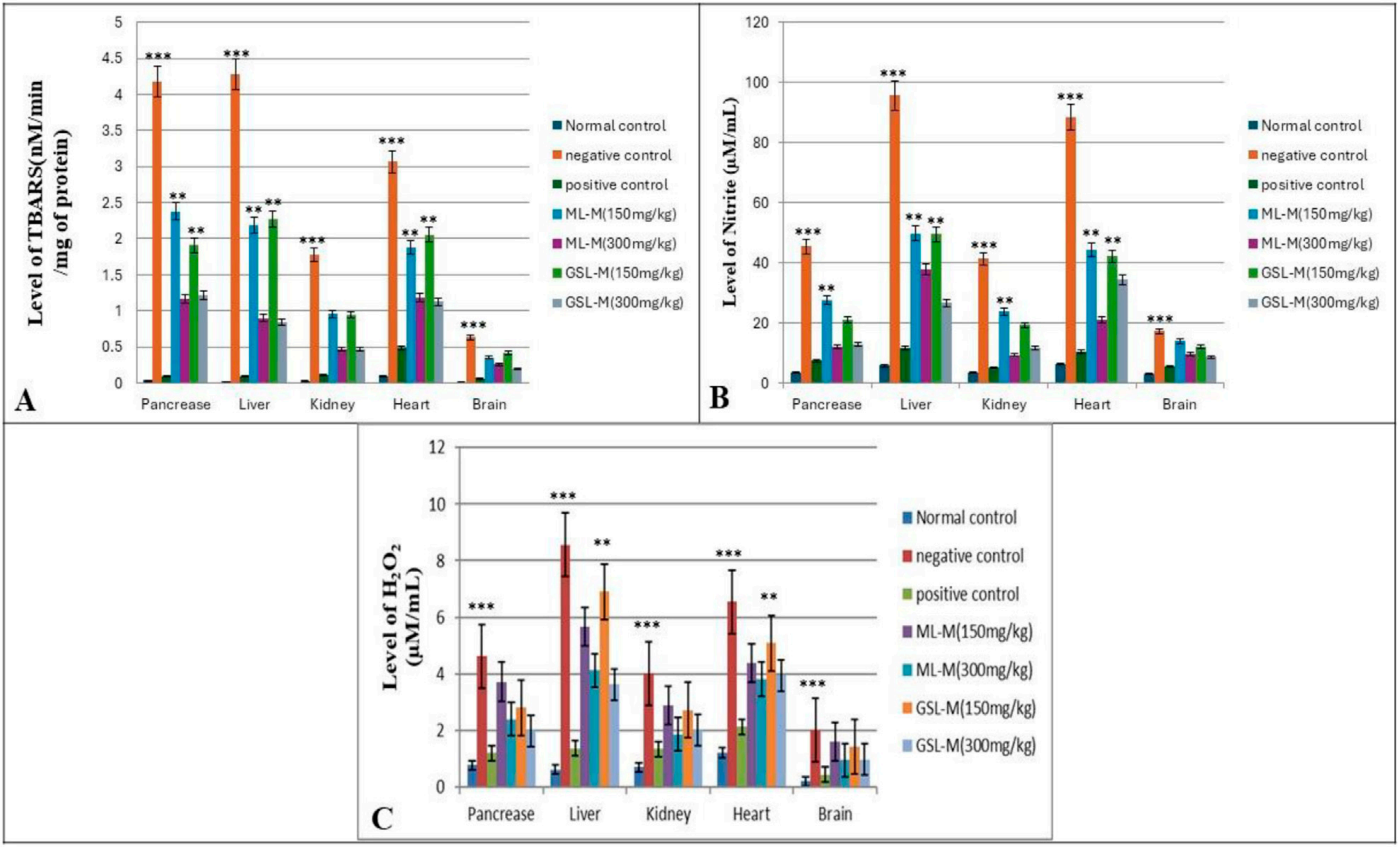
Effect of 150 mg/kg and 300 mg/kg doses of ML-M and GSL-M methanolic extracts on the status of oxidative stress markers in the pancreas, liver, kidney, heart, and brain tissues of diabetic animals. **(A)** Level of TBARS (nM/min/mg of protein). **(B)** Level of H_2_O_2_ (µM/mL). **(C)** Level of nitrite (µM/mL). Normal control: Non-diabetic, healthy animals. Negative control: Untreated, diabetic animals. Positive control: Glibenclamide-treated diabetic animals. ML-M: Marathon leaves methanol extract treated diabetic animals. GSL-M: Green sprout leaves methanol extract treated diabetic animals. All groups were compared to normal non-diabetic groups using one-way ANOVA followed by Tukey’s comparison. *P ≤ 0.05, **P ≤ 0.01, ***P ≤ 0.001.

The status of oxidative stress markers, including TBARS content, Nitrite, and H_2_O_2_ accumulation, was checked in all experimental groups. In the negative control, TBARS was significantly high, which is a clear indication of the occurrence of lipid peroxidation and, thus, oxidative stress in this group (Figure 3A). Increased level of lipid peroxidation is indirect evidence of intensified free-radical production and known to be involved in neuro, nephron, and hepatotoxicity (Repetto et al., 2012). Plant extract-treated groups showed a moderate effect in reversing the levels of TBARS in diabetic animals upon treatment as compared to standard drugs, where GSL-M at the concentration of 300 mg/kg appeared to be the most effective treatment for all cell types. ML-M at its high concentration (300 mg/kg) showed moderate to low activity to reverse TBARS content compared to GSL-M at similar concentrations. The results were consistent for all organs. Elevated concentration of H_2_O_2_ is directly linked with insulin signalling. It is involved in glucose uptake by stimulating GLUT4 receptors by adipocytes and muscles, the same event in oxidative stress (Pitocco et al., 2010). In our experiment, the levels of H_2_O_2_ were very high in the negative control group compared to the normal and positive control groups (Figure 3B), which shows the potential of alloxan to induce oxidative stress and affect all cell types. Broccoli plant extracts were significantly effective in controlling the levels of H_2_O_2_, GSL-M (300 mg/kg) being the most effective treatment. Nitrite, a cell nitric oxide metabolism product, is involved in oxidative stress, even in millimolar concentrations (May et al., 2004). In diabetic animals, the nitrite level was significantly higher than in normal control animals (Figure 3C). Treatment with the plant extracts lowered the elevated nitrite levels in all cell types (organs) in a dose-dependent manner. The alleviative effect of plant extract treatments in reducing the level of oxidative stress damage by decreasing TBARS, nitrite, and H_2_O_2_ levels, coincided with enhanced activities of antioxidant enzymes CAT, POD, and SOD and enhanced content of GSH in the pancreas, liver, kidney, heart, and brain tissues of diabetic animals in the present study.

### 3.5 Effect of ML-M and GSL-M on histo-architecture of vital organs (pancreas, liver, sKidney, heart, brain)

**FIGURE 4:**
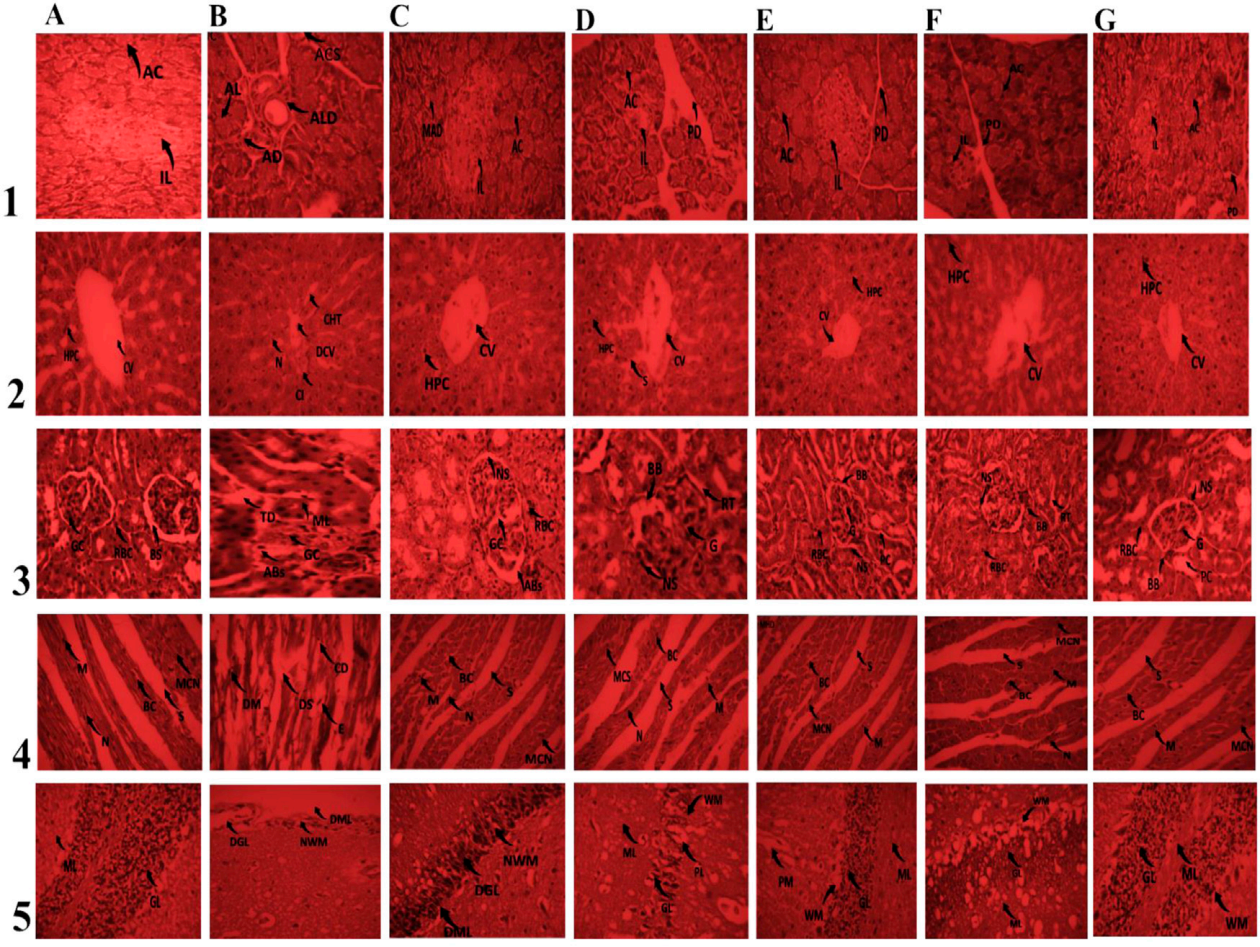
Effect of 150 mg/kg and 300 mg/kg doses of ML-M and GSL-M methanolic extracts on histo-architecture of vital organs. Column A: Normal control. Column B: Negative control. Column C: Positive control. Column; D ML-M 150 mg/kg. Column E; ML-M 300 mg/kg. Column F; GSL-M 150 mg/kg. Column G; GSL-M 300 mg/kg. Row (1) Pancreas; IL: islets of Langerhans; AC: Acinar cells; MLD: mild Langerhans disruption; ALD: acute Langerhans disruption; LD: Langerhans disruption; AD: acinar disintegration; ACS: acinar cell steatosis; IC: inflammatory cells; PD: pancreatic duct. Row (2) Liver; CV: central vein; HPC: hepatocytes; DCV: damaged central vein; CT: cellular infiltration; CHT: cellular hypertrophy; N: necrosis; S: sinusoids. Row (3) Kidney; G: glomerulus; BC: Bowman’s capsule; BS: Bowman’s space; ML: mild lobulation; CI: cellular infiltration; CD: capsule distortion; Abs: alterations in Bowman’s space; TD: tubule dilation; PT: proximal convoluted tubule; DCT: dilated convoluted tubule; RBC: regenerating Bowman’s capsule; LBB: loss of brush border; DCG: degenerative changes in glomerulus; RT: renal tubule; BB: brush border. Row (4) Heart; M: myocytes; DS: damaged striations; N: nucleus; E: edema; MCN: muscle cell nucleus; BC: blood capillary; CD: capillary damage; S: striation. Row (5) Brain; PM: Pia Matter; ML: Molecular Layer; GL: Granular Layer; WM: White Matter; PL: Purkinge’s cell Layer; NWM: Narrowed White Matter; GL: Degenerated Granular Layer; DML: Distorted Molecular Layer; WMN: White matter narrowed; MDPL: Mild Distorted Purkinge’s cell Layer; MDGL: Mild Distorted Granular Layer.

Histopathological studies revealed the protective effect of ML-M and GSL-M against alloxan-induced tissue damage. In the untreated diabetic control (negative control), pancreatic cells suffered acute disruption of Islets of Langerhans (IL) due to acinar cell (AC) steatosis (Figure 4, row 1). However, when treated with a standard drug, it significantly restored the normal structure with only mild cell disintegration (Figure 4, row 1, column C). ML-M and GSL-M dose-dependently reversed the pathological alterations induced by alloxan. ML-M and GSL-M protected islets of Langerhans from disintegration with a completely intact pancreatic duct (Figure 4, row 1, column D-G). Islets of Langerhans have stable cells that possess the ability to regenerate and can survive oxidative stress up to some extent. These cells can proliferate and replace the worn-out cells (Chakravarthy et al., 1980). The protective effect of ML-M and GSL-M could be due to the presence of insulin-like substances that promote the regeneration capacity of pancreatic cells.

Similar results were observed when the histo-architecture of the liver was examined. In negative control animals, inflammatory cell infiltrations, cellular hypertrophy, ballooning, and dilation of the central vein were the most prominent features observed (Figure 4, row 2, column B). Treatment with ML-M and GSL-M protected the central vein from improper dilation and reduced cellular hypertrophy dose-dependently (Figure 4, row 2, column D-G). GSL-M at its high dose showed more protective potential than any other treatment (Figure 4, row 2, column G). Alloxan-induced hypoglycemia and oxidative stress are the major known culprits behind hepatic cell damage (Ragavan and Krishnakumari, 2006). Treatment with ML-M and GSL-M significantly lowered the blood glucose, protecting the tissues against hyperglycemia-induced damage.

In the case of kidney tissues, both cortex and medulla were carefully examined for any tissue damage in all treated groups and normal animals. The negative control showed deleterious effects in renal tissues. Severe damage of cortical tissues was observed in this group due to oxidative stress induced by alloxan. The renal sections of this group showed severe impairments such as tubular dilation, tubular deterioration, glomerular atrophy, glomerular hypertrophy and obliteration of Bowman’s capsule of nephrons, clogging in capillaries tubule dilation, alterations in Bowman’s space and mild lobulation in diabetic control (Figure 4, row 3, column B). All these disturbances have been reported earlier in people with diabetes (El-Demerdash et al., 2005). Treatment with a high dose of ML-M and GSL-M noticeably conserved the normal morphology of the kidney, while a low dose of ML-M and GSL-M narrowed the chronic damages (Figure 4, row 3, column D-G).

Damaged heart muscles with distorted capillaries, degenerated muscle fibers, sub-endocardial necrosis, and edema were seen in animals used as negative control (Figure 4, row 4, column B) as compared to normal control where normal cardiac muscles with a central nucleus (distinct) and refined blood capillaries were obvious (Figure 4, row 4, column A). Similar conditions were observed in a previous study (Yin et al., 2018). In ML-M and GSL-M treated groups, myofibrils with continued striations were observed with branched appearance, intact endocardium, and pericardium represented standard architecture with no signs of inflammatory cell infiltration (Figure 4, row 4, column D-G).

In the normal group, proper layers, i.e., the outer layer (molecular layer), middle layer (Purkinje cell layer), and the inner layer (granular layer) of the cerebellum along with pia matter, were clearly visible (Figure 4, row 5, column A). Alloxan-treated groups revealed distortion in the histo-architecture of the narrowed white matter, cerebellum, degenerated granular layer, loss of Purkinje’s cell layer, and distorted molecular level (Figure 4, row 5, column B). Agbon et al. (2014) previously documented similar deterioration. The control group treated with the drug exhibited mild alterations. While treatment with a high dose of broccoli cultivars exhibited an effect close to normal, and a low dose of ML-M and GSL-M also showed a protective effect (Figure 4, row 5, column D-G).

### 3.6 HPLC analysis of quercetin and kaempferol in green and etiolated leaves

**FIGURE 5:**
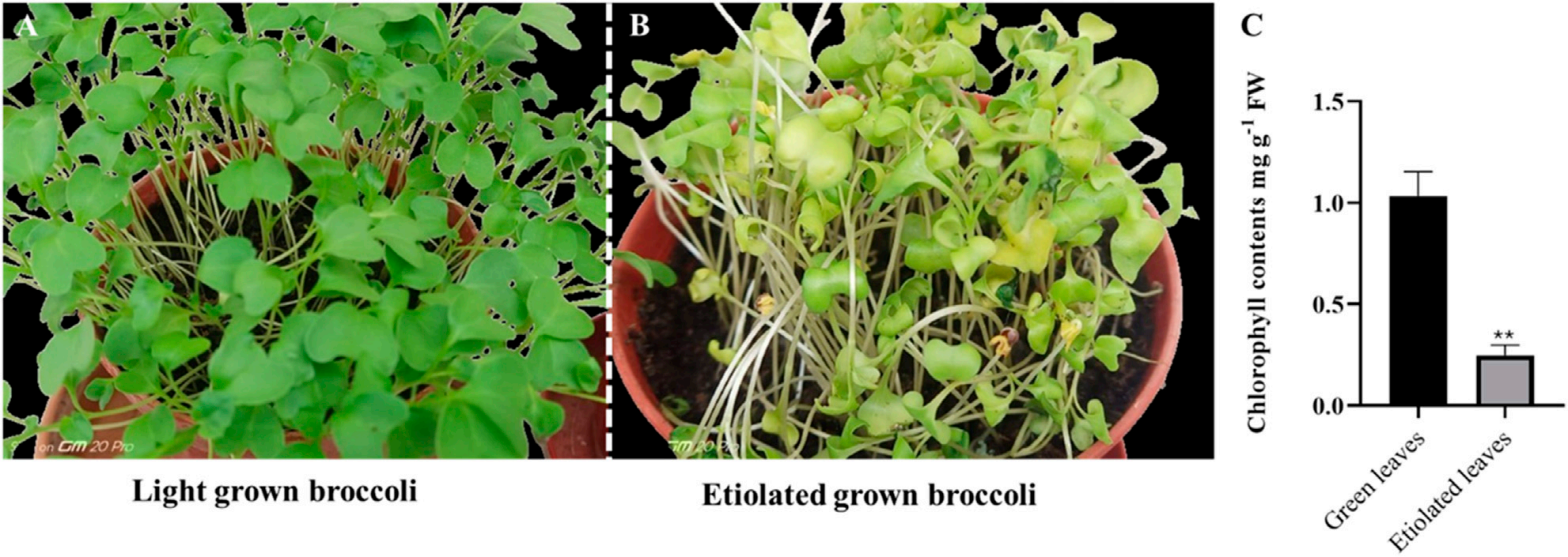
Phenotype and chlorophyll contents of light and dark (etiolated) grown seedlings. The broccoli seedlings were grown under light **(A)** and dark **(B)** for 2 weeks. The chlorophyll contents were measured after 2 weeks **(C)**. Asterisks show a significance level of *P* < 0.01. Data shows an average of three repeats; error bars represent ± SE.

In humans, flavonoids are associated with a wide range of health benefits due to their bioactive properties, including anti-inflammatory, anti-cancer, anti-aging, cardioprotective, neuroprotective, immunomodulatory, anti-diabetic, antibacterial, anti-parasitic and antiviral properties (Alkhalidy et al., 2018; Sok Yen et al., 2021). In plants, these compounds are involved in various activities such as cell growth regulation, protecting plants against biotic and abiotic environmental factors, attracting pollinators, etc., (Agati et al., 2012). Kaempferol and quercetin are the main class of flavonols and differ in the OH group, with quercetin having an extra OH group on the third position of the B-ring. They are abundant in onions, apples, and broccoli and have great therapeutic potential for human health (Jan et al., 2022). After having seen both the *in vitro* and *in vivo* antidiabetic and protective effects of broccoli extracts in reducing the level of oxidative stress-induced tissue damage in this study, we further explored the accumulation pattern of flavonoid content (quercetin and kaempferol) in leaves under control and etiolated conditions. For this purpose, broccoli seedlings were grown under light (16 h light/8 h dark) (Figure 5A) and continuous dark (Figure 5B) for 7 days. For chlorophyll contents, plants were exposed to constant darkness for 2 weeks, and chlorophyll levels were significantly lower in etiolated leaves than in those grown under light conditions (Figure 5C).

**FIGURE 6:**
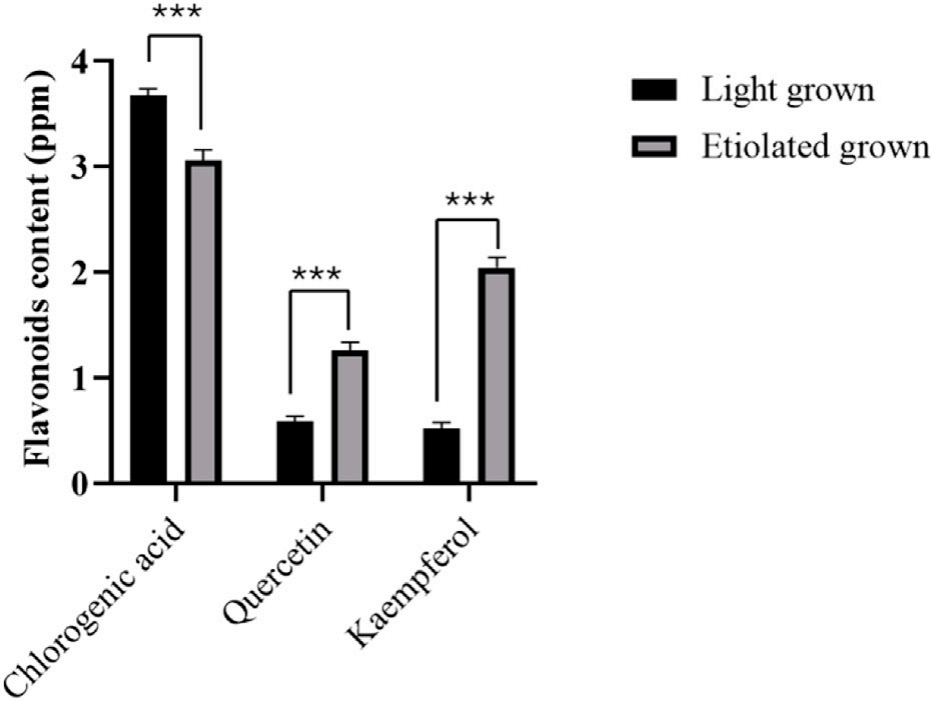
Flavonoids level in broccoli seedlings. Chlorogenic acid, quercetin, and kaempferol were measured from 15-day-old seedlings grown in light and dark conditions for 7 days. Data represented the mean ± SE of three replications. Asterisks show a significance level of *P* < 0.001.

The HPLC-based quercetin and kaempferol levels were significantly higher in broccoli seedlings’ etiolated leaves (Figure 6). However, chlorophyll content decreased in etiolated leaves, suggesting the synthesis and accumulation of quercetin and kaempferol but chlorophyll degradation under dark conditions. On the other hand, chlorogenic acid content was significantly lower in etiolated leaves than in green leaves. Our results corroborate with the previous findings in tea leaves, where quercetin and kaempferol were enhanced in albino leaves than in green leaves (Zhang et al., 2022), suggesting the evolutionarily conserved biosynthesis and accumulation mechanism of quercetin and kaempferol in tea and broccoli leaves.

On the other hand, the accumulation of flavonoids and carotenoids but chlorophyll degradation was reported to promote the yellowing of leaves in *Ginkgo biloba* (Shi et al., 2012) and macadamia (Yang et al., 2023). This might also be the case in etiolated broccoli leaves, whose chlorophyll content showed a decline, but flavonoids like quercetin and kaempferol increased in the present study.

### 3.7 Real-time qPCR analysis of ABC transporter in light and dark-grown seedlings

**FIGURE 7:**
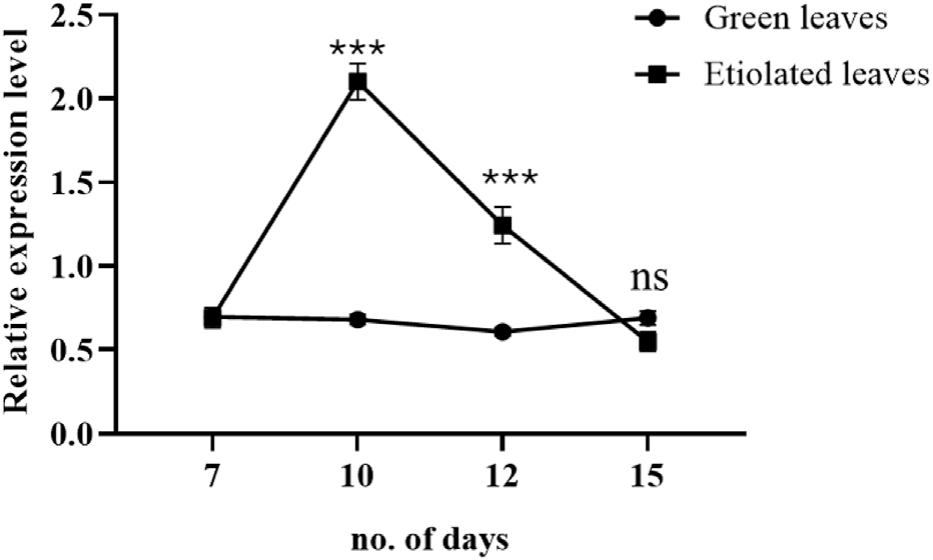
Effects of dark and light on the expression level of broccoli ABC transporter (BolC8t51326H). The dark condition was imposed on the day 7th of the broccoli seedlings. The expression was determined during days 7, 10, 12, and 15 of growing broccoli seedlings in light or dark. Data represent the mean ± SE of three independent biological repeats. Asterisks show a significance level of *P* < 0.001.

Three-day exposure of broccoli seedlings to dark conditions significantly upregulated the expression level of the ABC transporter at the growth stage on day ten compared to light-grown seedlings. After 5-day exposure to darkness (on the 12th day of the growing stage), the expression level was decreased but still significantly higher than the light-grown seedlings. However, on the 15th day of the growing stage, the expression level of the ABC transporter was downregulated to the level of light-grown seedlings (Figure 7).

### 3.8 Phylogenetic tree, subcellular localization, and membrane topology

**FIGURE 8:**
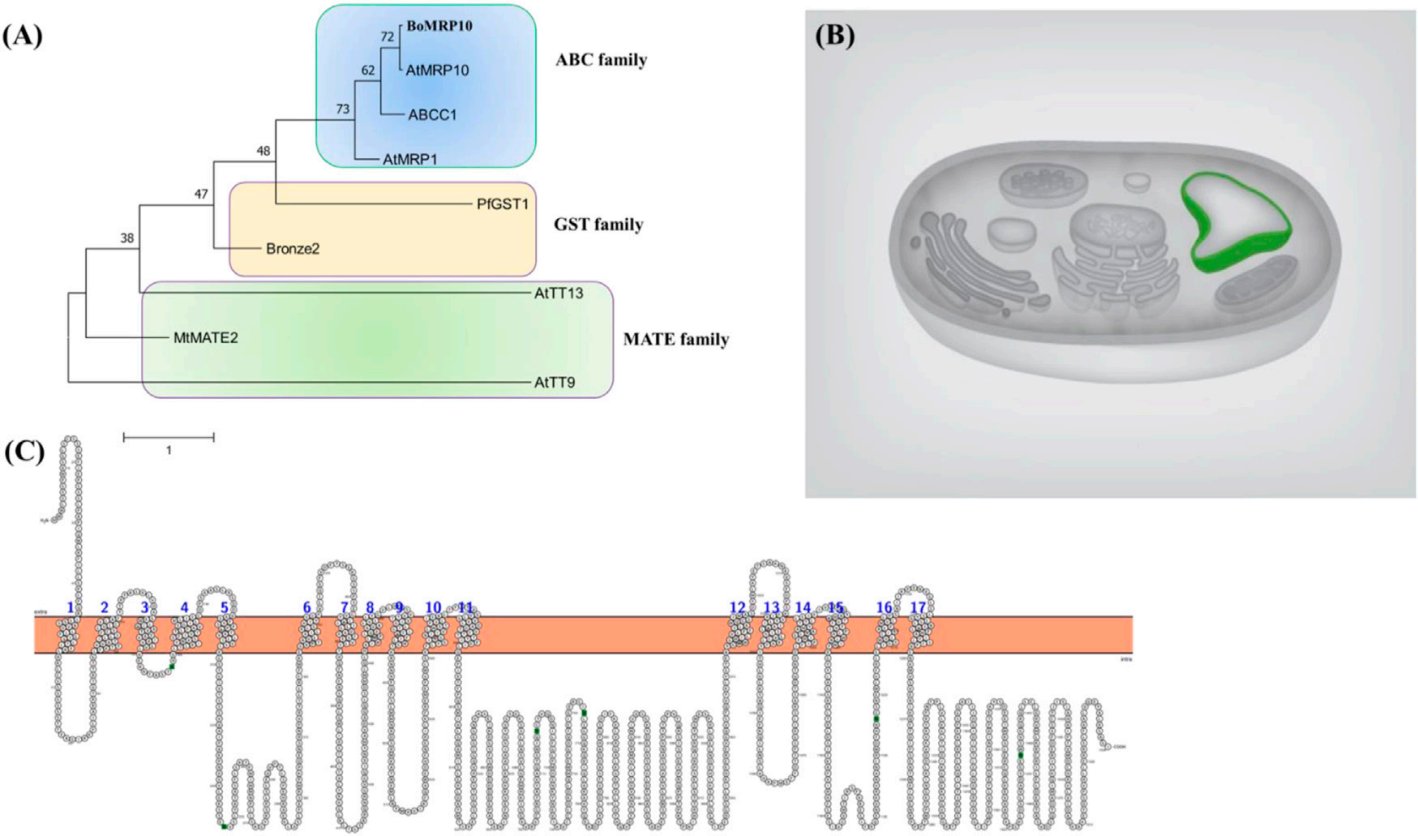
Phylogenetic tree construction **(A)**, predicted subcellular localization **(B)**, and membrane topology **(C)** of BoMRP10 (BolC8t51326H). The protein accessions used were BoMRP10 (BolC8t51326H), Bronze2 (X81971.1; Marrs et al., 1995), PfGST1 (AB362191; Yazaki et al., 2008), ABCC1 (JX245004; Francisco et al., 2013), AtMRP1 (AF008124; Lu et al., 1998), AtTT9 (At3g28430; Ichino et al., 2014), AtMRP10 (At3G62700; Sugiyama et al., 2006), AtTT13 (At1g17260; Appelhagen et al., 2015), MtMATE (HM856605.1; Zhao et al., 2011). Prediction shows tonoplast localization of the transporter and 17 membrane helices across the membrane.

The phylogenetic tree was constructed using the neighbor-joining method. The broccoli transporter grouped in ABC family protein transporters and closely resembled AtMRP10; therefore, we named it BoMRP10 ABC transporter (Figure 8A). The ABC transporter protein sequence was used to predict its cellular localization. The protein was localized to the vacuolar membrane (Figure 8B), indicating the putative accumulation role of quercetin and kaempferol in vacuoles of broccoli leaves. The membrane topology of the transporter showed 17 transmembrane helices (Figure 8C). In a recent study, the functional role of Yeast Cadmium Factor 1 (Ycf1) belonging to the ATP binding cassette C-subfamily (ABCC) of transporters and sequesters glutathione into vacuole has been shown to have 17 transmembrane helices (Khandelwal et al., 2022). The ABC transporter in this study having highly similar subcellular localization and membrane topology, shows a significant role in the accumulation of flavonoids like quercetin and kaempferol in broccoli leaves.

Although several gene families of transporters such as Glutathione S-transferases (GST), Multidrug resistance-associated protein (MRP), and multi-drug and toxic compound extrusion (MATE) are involved in transporting flavonoids (Manzoor et al., 2023) into vacuolar accumulation processes, however, in this study we focused on broccoli ABC transporter which is a superfamily protein (Klein et al., 2006). The transporters localized to the tonoplast are reported responsible for the accumulation of flavonoids in various plant species. *AtTT9* gene from the MATE family, although localized to the Golgi apparatus, is involved in vacuolar development. Therefore, mutants of the *AtTT9* reduced the level of kaempferol and quercetin, including other flavonoids in the seed coat, suggesting its role in facilitating flavonoids accumulating into vacuoles (Ichino et al., 2014). Flavonoids, including kaempferol and quercetin, are reported to be accumulated into the vacuole by GST, MATE, and ABC transporters (Zhao, 2015; Ku et al., 2020).

**FIGURE 9:**
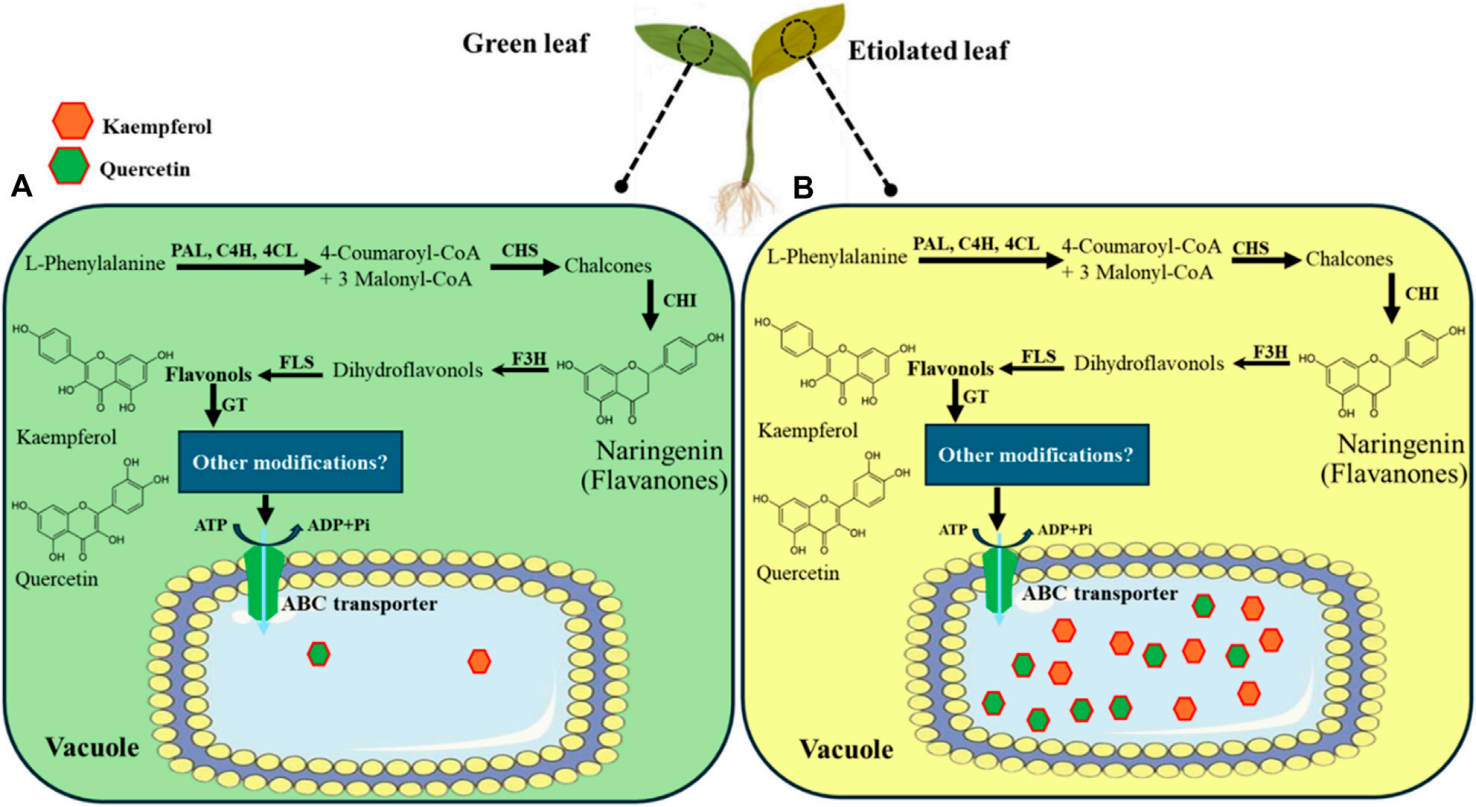
Pathway for kaempferol and quercetin synthesis, transport, and accumulation into vacuole via vacuolar ABC transporter in broccoli. The pathway was modified and adapted from Zhao et al. (2011). The dark condition was imposed on the 7th day of the broccoli seedlings, and green **(A)** and etiolated **(B)** leaves were sampled on the 15th day. The 7-day continuous dark condition triggered a higher accumulation of flavonoids such as kaempferol and quercetin in etiolated leaves than in light-grown seedlings.

Our findings clearly demonstrated that antidiabetic flavonoids like kaempferol and quercetin accumulated in etiolated seedlings than in green seedlings. Chlorophyll is responsible for the greening of leaves (Figure 5). However, a decrease in chlorophyll content could, therefore, trigger the generation of kaempferol and quercetin as a defence mechanism to manage the cellular stress caused by the lack of chlorophyll. A recent report also supports our study that the accumulation of kaempferol and quercetin in albino leaves resulted from oxidative stress (Zhang et al., 2022). Thus, our study further postulates a novel way for the enhanced production of flavonoids by reducing chlorophyll content in plants, especially in broccoli leaves, as illustrated in Figure 9.

## 4 Conclusion

In conclusion, both plant extracts showed a dose-dependent inhibition of alloxan-induced hyperglycaemic oxidative damage, which suggests their role as a potential natural antidiabetic agent. Study demonstrates that the complete absence of light favours the enhanced accumulation of kaempferol and quercetin, and the expression of the putative ABC transporter *BoMRP10* was closely connected to their accumulation. Taken together, our study postulates a novel method for the enhanced accumulation of kaempferol and quercetin as antidiabetic agents.

## 5 Future perspectives

In future, the putative ABC transporter should be elaborated functionally by electrophysiology for its transport of compounds like kaempferol and quercetin in isolated oocytes, subcellular localization in protoplast and also genome editing of this transporter in broccoli to test its functional activity for accumulation of the compounds.

## Data Availability

The raw data supporting the conclusions of this article will be made available by the authors, without undue reservation.

## Abbreviations

GSL-M, Methanolic extract of cv. Green Sprout; ML-M, Methanolic extract of cv. Marathon.
